# Coverage of clinic-based TB screening in South Africa may be low in key risk groups

**DOI:** 10.5588/pha.15.0064

**Published:** 2015-01-25

**Authors:** N. McCreesh, I. Faghmous, C. Looker, P. J. Dodd, I. D. Plumb, K. Shanaube, M. Muyoyeta, P. Godfrey-Faussett, H. Ayles, R. G. White

**Affiliations:** 1 TB Modelling Group, Department of Infectious Disease Epidemiology, London School of Hygiene & Tropical Medicine (LSHTM), London, UK; 2 Health Economics and Decision Science, School of Health and Related Research, University of Sheffield, Sheffield, UK; 3 ZAMBART Project, School of Medicine, University of Zambia, Lusaka, Zambia; 4 TB Department, Centre for Infectious Disease Research in Zambia, Lusaka, Zambia; 5 Department of Clinical Research, LSHTM, London, UK

**Keywords:** tuberculosis, screening, South Africa, primary health care

## Abstract

The South African Ministry of Health has proposed screening all clinic attendees for tuberculosis (TB). Amongst other factors, male sex and bar attendance are associated with higher TB risk. We show that 45% of adults surveyed in Western Cape attended a clinic within 6 months, and therefore potentially a relatively high proportion of the population could be reached through clinic-based screening. However, fewer than 20% of all men aged 18–25 years, or men aged 26–45 who attend bars, attended a clinic. The population-level impact of clinic-based screening may be reduced by low coverage among key risk groups.

## Abstract (FR)

Le Ministère de la Santé d'Afrique du Sud a proposé de dépister la tuberculose (TB) chez tous les patients visitant un centre de santé. Parmi d'autres facteurs, le sexe masculin et la fréquentation des bars sont associés à un risque plus élevé de TB. Nous montrons que 45% des adultes dépistés dans la province du Cap Ouest s'étaient rendus dans un centre de santé au cours des 6 derniers mois et c'est pourquoi une proportion relativement élevée de la population pourrait être atteinte à travers un dépistage en centre de santé. Cependant, moins de 20% de tous les hommes âgés de 18–25 ans, ou des hommes âgés de 26–45 ans qui fréquentent les bars, se rendent dans un centre de santé. L'impact sur la population de ce type de dépistage pourrait donc être réduit par une faible couverture parmi les groupes à risque majeur.

## Abstract (ES)

El Ministerio de Salud de Suráfrica propuso una detección sistemática de la tuberculosis (TB) a todas las personas que acudían a los consultorios. Entre los factores asociados con un mayor riesgo de padecer TB están el sexo masculino y la frecuentación de bares. El presente artículo pone de manifiesto que 45% de los adultos encuestados en la Ciudad del Cabo había acudido a un establecimiento de salud en los últimos 6 meses, por lo cual se pudo llegar a una proporción relativamente alta de la población mediante esta detección sistemática. Sin embargo, menos del 20% de todos los hombres entre los 18 y los 25 años, o entre los 26 y los 45 años de edad que frecuenta los bares, acudió a los establecimientos de salud. La repercusión a escala de la población de una detección sistemática realizada en los consultorios podría verse atenuada por una baja cobertura de los grupos más vulnerables.

South Africa has one of the highest tuberculosis (TB) burdens in the world, with an estimated disease prevalence in 2013 of 715 per 100 000 population.^1^ Disease incidence has been falling since 2008,^2^ but the decline is slow, and new approaches are needed to accelerate the rate of decline. As part of its ‘90s’ strategy, on World TB Day 2015, the South African Ministry of Health proposed implementing screening of all health centre attendees for TB disease (unpublished speech given by Minister of Health A. Motsoaledi). This is a potentially more cost-effective approach than community-based screening programmes, as it is plausible that the prevalence of TB will be higher in clinic attendees than in the wider community, and the costs of reaching clinic attendees are likely to be lower than more active community-based screening programmes. Its population-level impact will be lower, however, if screening coverage is low among population subgroups that are at higher risk of developing TB disease. We investigated clinic attendance in adults in Western Cape, and in particular among two key risk groups: (young) men and people who visit bars.

## STUDY POPULATION, DESIGN AND METHODS

The study sampling frame consisted of adults living in eight communities in Western Cape, South Africa, who were enrolled in the final Zambia/South Africa TB and acquired immune-deficiency syndrome Reduction (ZAMSTAR) prevalence survey.^3^ Four sampling enumeration areas (SEAs) from each ZAMSTAR community were selected proportional to size, and 40 people were sampled at random from each SEA, stratified by sex and age. A full description of the sampling method is given elsewhere.^4^

Respondents were interviewed in their own homes using a structured questionnaire.^4^ The main purpose of the questionnaire was to collect data on contact and activity patterns relevant to Mycobacterium tuberculosis transmission. Respondents were also asked if they had visited a health care facility for any reason in the last 6 months.

Logistic regression was used to examine associations between a number of variables and clinic attendance. Estimates were adjusted for age and sex and weighted to account for the stratified sampling design.

Ethical approval was obtained from the University of Stellenbosch Health Research Ethics Committee, Stellenbosch, South Africa (N04/10/173), the University of Zambia Biomedical Research Ethics Committee, Lusaka, Zambia (007-10-04), and the London School of Hygiene & Tropical Medicine Ethics Committee, London, UK (A211 3008).

## RESULTS

A total of 1272 adults living in Western Cape were interviewed. Results are presented for the 1271 adults who provided information on clinic attendance.

Overall, 55% (95% confidence interval [CI] 48–61) of women and 29% (95%CI 23–34) of men reported visiting a clinic in the past 6 months (Table 1). Adjusting for sex, the odds of having visited a clinic increased with age (*P* = 0.001), with people aged 18–25 years having 0.47 (95%CI 0.31–0.70) times the odds compared to people aged >45 years. People who reported attending bars had 0.45 (95%CI 0.33–0.60) times the odds of clinic attendance compared to people who reported never attending bars.

**TABLE 1:**
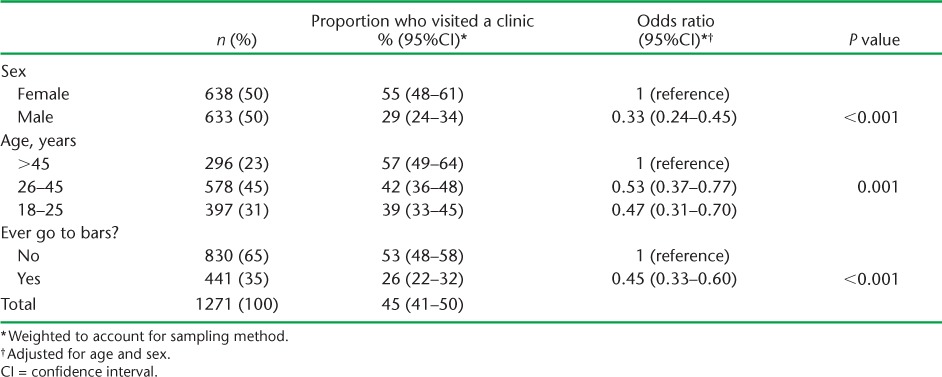
Association between sex, age and bar attendance and clinic attendance within the previous 6 months

Reported clinic attendance in the past 6 months was >50% for women in all age groups who never attended bars and for women aged >45 years who attended bars (Table 2). Reported attendance was <20% for all men aged 18–25 and for men aged 26–45 who attended bars.

**TABLE 2:**
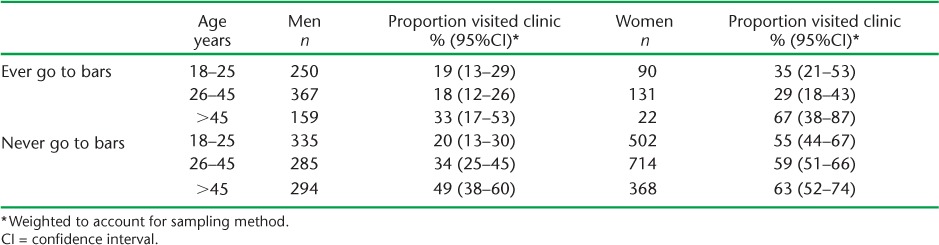
Proportion of people reporting clinic attendance in past 6 months by sex, age and bar attendance

## DISCUSSION

We demonstrate that a clinic-based screening approach in Western Cape could achieve a potentially relatively high overall level of population coverage, with 45% of adults reporting visiting a clinic in the last 6 months. In line with other studies from South Africa, we show that clinic attendance is lower among men than women,^5^,^6^ and is lower in younger than older adults.^5^ Coverage may be substantially lower than average in some population groups, in particular men aged 18–25 and men aged 26–45 who visit bars. Fewer than 20% of men in these groups may be reached by clinic-based screening programmes in any 6-month period. This is of particular concern, as these demographic groups are at increased risk of contracting TB,^7^,^8^ and passive case detection may be particularly inadequate in people who consume alcohol,^9^ potentially reducing the wider impact of clinic-based TB screening programmes.

We present data on clinic visits in the last 6 months. Coverage will be higher over longer time periods, as a higher proportion of the population will visit a clinic in 12 months, for example, than in 6 months. For people who visit clinics less frequently, however, time between screenings will be longer, reducing the average impact of screening on the time between the onset of infectiousness and diagnosis. Coverage will also be lower if not all clinics screen 100% of attendees.

The main limitation of our approach is that data on both clinic and bar attendance were collected for similar time periods. This means that some of the observed inverse association between bar and clinic attendance may have resulted from illness that caused individuals to both attend a clinic and to stop attending bars, rather than a genuine reluctance or inability to attend clinics in people who visit bars. This may have biased the association upwards, but is unlikely to be responsible for more than a small proportion of the large observed difference in clinic attendance.

A second limitation of our findings is that only communities with a clinic could be selected for inclusion in the original ZAMSTAR study. Clinic usage has been shown to decrease in South Africa as distance to the nearest clinic increases.^10^ Coverage may therefore be lower in communities with poorer access to health care, reducing overall coverage.

Clinic-based TB screening programmes have the potential to reach nearly half the adult population over a 6-month period in Western Cape, and may be a cost-effective way to reduce the prevalence of TB. Their effectiveness may be reduced by poor coverage in certain risk groups, however, and we recommend that clinic-based screening should be supplemented by case-finding programmes targeting key risk groups.
